# Exome Sequencing Identifies a Rare *HSPG2* Variant Associated with Familial Idiopathic Scoliosis

**DOI:** 10.1534/g3.114.015669

**Published:** 2014-12-12

**Authors:** Erin E. Baschal, Cambria I. Wethey, Kandice Swindle, Robin M. Baschal, Katherine Gowan, Nelson L.S. Tang, David M. Alvarado, Gabe E. Haller, Matthew B. Dobbs, Matthew R.G. Taylor, Christina A. Gurnett, Kenneth L. Jones, Nancy H. Miller

**Affiliations:** * Department of Orthopedics, University of Colorado Denver Anschutz Medical Campus, Aurora, Colorado, 80045; † Musculoskeletal Research Center, Children’s Hospital Colorado, Aurora, Colorado, 80045; ‡ Department of Biochemistry, University of Colorado Denver Anschutz Medical Campus, Aurora, Colorado, 80045; § Department of Chemical Pathology and Li Ka Shing Institute of Health Sciences, Faculty of Medicine, The Chinese University of Hong Kong, Hong Kong SAR; ** Department of Orthopaedic Surgery, Washington University School of Medicine, St. Louis, Missouri, 63130; †† Saint Louis Shriners Hospital for Children, St. Louis, Missouri, 63131; ‡‡ Department of Cardiology, University of Colorado Denver Anschutz Medical Campus, Aurora, Colorado, 80045; §§ Department of Neurology, Washington University School of Medicine, St. Louis, Missouri, 63130

**Keywords:** *HSPG2*, perlecan, idiopathic scoliosis, exome sequencing

## Abstract

Idiopathic scoliosis occurs in 3% of individuals and has an unknown etiology. The objective of this study was to identify rare variants that contribute to the etiology of idiopathic scoliosis by using exome sequencing in a multigenerational family with idiopathic scoliosis. Exome sequencing was completed for three members of this multigenerational family with idiopathic scoliosis, resulting in the identification of a variant in the *HSPG2* gene as a potential contributor to the phenotype. The *HSPG2* gene was sequenced in a separate cohort of 100 unrelated individuals affected with idiopathic scoliosis and also was examined in an independent idiopathic scoliosis population. The exome sequencing and subsequent bioinformatics filtering resulted in 16 potentially damaging and rare coding variants. One of these variants, p.Asn786Ser, is located in the *HSPG2* gene. The variant p.Asn786Ser also is overrepresented in a larger cohort of idiopathic scoliosis cases compared with a control population (*P* = 0.024). Furthermore, we identified additional rare *HSPG2* variants that are predicted to be damaging in two independent cohorts of individuals with idiopathic scoliosis. The *HSPG2* gene encodes for a ubiquitous multifunctional protein within the extracellular matrix in which loss of function mutation are known to result in a musculoskeletal phenotype in both mouse and humans. Based on these results, we conclude that rare variants in the *HSPG2* gene potentially contribute to the idiopathic scoliosis phenotype in a subset of patients with idiopathic scoliosis. Further studies must be completed to confirm the effect of the *HSPG2* gene on the idiopathic scoliosis phenotype.

Idiopathic scoliosis (IS) [MIM: %181800] is a common inherited disorder of the immature skeleton, affecting 3% of the pediatric population, with girls characteristically more severely affected than boys (Asher and Burton 2006). Clinically, IS is defined as a structural lateral spinal curvature of ≥10° with a rotatory component, documented by radiographic analysis and occurring in otherwise-normal children. Current treatment modalities are limited to observation, physical therapy, bracing, and surgery. Significant curves in adolescence can lead to severe curve progression in adulthood. In an effort to prevent severe progression and deformity as an adult, these adolescents may elect to undergo an operative spinal instrumentation and fusion, a costly procedure with life-long implications. These methods focus on the physical symptoms and cosmetic deformity but fail to address the need for preventative options.

The familial nature of IS is well established, but the mode of inheritance is not clear (Cowell *et al.* 1972; Riseborough and Wynne-Davies 1973; Bonaiti *et al.* 1976; Czeizel *et al.* 1978; Tang *et al.* 2012). It is unlikely that IS families are homogeneous with respect to their genetic architecture but represent a spectrum of inheritance patterns ranging from simple Mendelian to complex polygenic. Concordance rates have been reported between 0.73 and 0.92 for monozygotic twins and between 0.36 and 0.63 for dizygotic twins (Kesling and Reinker 1997; Inoue *et al.* 1998; Andersen *et al.* 2007). Despite clear evidence for the importance of genetics in the susceptibility to IS, limited progress has been made in the identification of genes that are causative or may influence disease susceptibility, variation, or progression. The current literature includes multiple studies that have used linkage and association analyses. Genome-wide linkage analyses have effectively localized candidate regions; however, progress has been burdened by heterogeneity both within and between study populations and by insufficient statistical power (Wise *et al.* 2000; Chan *et al.* 2002; Salehi *et al.* 2002; Justice *et al.* 2003; Miller *et al.* 2005, 2006; Alden *et al.* 2006; Ocaka *et al.* 2008; Gurnett *et al.* 2009; Raggio *et al.* 2009; Clough *et al.* 2010; Marosy *et al.* 2010; Edery *et al.* 2011). Association studies, including genome-wide association studies (GWAS), have implicated select genes, including *CHD7*, *LBX1*, and *GPR126* (Gao *et al.* 2007; Sharma *et al.* 2011; Takahashi *et al.* 2011; Kou *et al.* 2013). A recent study has completed exome sequencing for an IS cohort and identified rare variants in the fibrillin genes, *FBN1* and *FBN2*, which were associated with curve severity (Buchan *et al.* 2014).

In the current study, we completed exome sequencing for a multigenerational family with familial IS, with the goal of identifying rare genetic variants that contribute to the IS phenotype.

## Material and Methods

### Subjects

#### Denver IS population:

For the Denver IS population, written informed consent was obtained from study subjects who were enrolled in accordance with protocols approved by the Johns Hopkins School of Medicine Institutional Review Board and the University of Colorado Denver Institutional Review Board (Colorado Multiple Institutional Review Board, Study Approval #06-1161 and 07-0417). A diagnosis of IS required clinical/family history and physical examination consistent with a spinal curvature in the coronal plane, a standing anteroposterior spinal radiograph showing ≥10° curvature by the Cobb method with pedicle rotation, and no congenital deformity or other existing genetic disorder (Shands and Eisberg 1955; Kane 1977; Armstrong *et al.* 1982). Individuals were classified as having familial IS if they had at least one additional family member affected with IS (Justice *et al.* 2003; Miller *et al.* 2005). The majority of individuals in our study are likely to exhibit an autosomal dominant mode of inheritance (Miller *et al.* 2005, 2012). We collected blood samples from all participants and extracted genomic DNA according to standard phenol-chloroform purification protocols (Sambrook *et al.* 1989; Moore and Dowhan 2002) or using the QIAGEN Gentra Puregene Blood Kit.

#### St. Louis IS population:

As described by Buchan *et al.* (2014), IS patients in the St. Louis dataset were recruited from St. Louis Children’s Hospital and St. Louis Shriners Hospital for Children. All patients had scoliosis of unknown etiology with spinal curves measuring ≥10°. Patients with developmental delay, multiple congenital anomalies, or known underlying medical disorders (*e.g.*, Ehlers-Danlos syndrome, Marfan syndrome) were excluded. We selected 140 unrelated IS cases of European ancestry with severe deformity (spinal curves measuring ≥40°) for the exome sequencing screen. DNA was collected from affected probands after we obtained informed consent.

#### Control populations:

Comparisons with control populations used either the 1000 Genomes population (European ancestry, N = 379) (Abecasis *et al.* 2012) or the National Heart, Lung, and Blood Institute (NHLBI) Exome Sequencing Project [(ESP) European ancestry subset (EA-ESP), version ESP6500SI-V2, N = 4300].

### Exome sequencing

#### Denver exome sequencing:

A multigenerational family with European ancestry was selected for exome sequencing, with DNA samples available on five affected and three unaffected individuals (two of the unaffected individuals are marry-in relatives). This family was selected based on the number of affected family members with severe curves and the relative pedigree-distance between affected family members. Exome capture was performed on 1 µg of genomic DNA from three affected individuals (IV:1, IV:6, and II:4) in this family using the Illumina TruSeq Exome Capture kit. Samples were sequenced with a 2x100bp run at the Illumina HiSeq 2000 at the University of Colorado Denver Genomics and Microarray Core Facility.

#### St. Louis exome sequencing:

For the St. Louis IS dataset, exon enrichment was performed using the SureSelect Human All Exon 38Mb and 50Mb kits (Agilent Technologies, Santa Clara, CA) or the TruSeq Exome Enrichment kit (Illumina, San Diego, CA) at the Washington University Genome Technology Access Center, as described previously (Buchan *et al.* 2014).

### Bioinformatics filtering

Exome sequencing reads were mapped to the reference human genome sequence (hg19) with large-scale alignment software (GSNAP) (Wu and Nacu 2010). Sequence calls for variants (single nucleotide variants (SNVs), insertions/deletions) were performed using the Broad Institute’s Genome Analysis Toolkit (GATK) (McKenna *et al.* 2010; Depristo *et al.* 2011). The resulting variants were filtered in a systematic way. The program ANNOVAR was used to filter the variants by cross referencing various genetic variation databases (*e.g.*, 1000 genomes database, NHLBI Exome Sequencing Project 6500 dataset, etc), allowing us to extract information about variant frequencies (if previously reported) and location within genes (Wang *et al.* 2010). Variants were restricted to those that 1) had no more than a 1% frequency in the phase 1 1000 genomes data (Abecasis *et al.* 2012), and 2) altered the coding sequence (nonsense, splice-site, missense and insertion/deletion). The nonsense and missense variants were subsequently cross-referenced to the dbNSFP database to determine whether the resulting changes to the protein are predicted to be damaging (see Supporting Information, File S1) (Liu *et al.* 2011). In addition, we required at least 5X sequence coverage at any given position to call a genotype. For the multigenerational family, we required that all three individuals share the variant if they had a genotype at that position.

For the St. Louis dataset, next-generation sequencing reads were aligned to hg19 human reference sequence and variants were called and annotated, as described by Buchan *et al.* (2014).

### Sanger sequencing

The variants that remained after the filtering process were confirmed by Sanger sequencing. The variants identified through exome sequencing were tested in the original three affected individuals who underwent exome sequencing and five additional relatives (two affected and three unaffected). As discussed in the *Results* section, one variant (chromosome 6, 168376925, in *HGC6.3*) had a high frequency in the EA-ESP population and therefore was not Sanger sequenced.

Primers were designed using the program Primer3 (Koressaar and Remm 2007; Untergasser *et al.* 2012). Primer sequences can be found in Table S1. Sanger sequencing was completed at either the University of Colorado Denver Barbara Davis Center for Childhood Diabetes or at Beckman Coulter Genomics. Details of PCR conditions and Sanger sequencing protocols are located in File S1.

Sanger sequences were analyzed using CodonCode software (CodonCode Corporation, Centerville, MA), and figures were generated using Sequencher (GeneCodes Corporation, Ann Arbor, MI).

### *HSPG2* sequencing

Exons from the *HSPG2* gene (97 exons, 14369 base pair mRNA, 4391 amino acids) were sequenced in 100 unrelated individuals with IS. All individuals had a curvature of >10° with a range of 18−119°), and were predominantly of European ancestry. To decrease sequencing costs, sequencing was completed using 25 pools of four individuals each. An Illumina MiSeq 2x250bp run was performed at the University of Colorado Denver DNA Diagnostic Laboratory. The *HSPG2* libraries were enriched using long-range polymerase chain reaction (PCR), resulting in approximately 2.5 to 10 kb fragments with a Takara long range PCR kit (Clontech). PCR conditions followed manufacturer’s recommendations. Primer sequences can be found in Table S1. For high GC regions, betaine was used in the PCR. A GENios FL (Tecan) microplate reader using SYBR green was used to quantify the libraries. The library was generated using a Nextera XT DNA sample kit (FC-121-1031; Illumina) and Nextera Index kit (FC-121-1011; Illumina).

Bioinformatics filtering was completed using the same protocol as described earlier. The filtering process resulted in 14 potentially damaging and rare *HSPG2* variants in these 100 individuals. The 14 variants were Sanger sequenced in each of the four individuals within the pool to confirm the variant and identify the individual within the pool that carried the variant. The Sanger sequencing protocol was the same as described for the multigenerational family. We were unable to confirm an *HSPG2* variant at position 22263709 (p.Met1Thr) due to problems with the PCR-amplification process. This variant was removed from all further analyses, resulting in a total of 13 rare variants in the *HSPG2* gene.

The median sequencing coverage across the *HSPG2* gene was 172X, with a range of 45X to 349X. Exons 27−42 had lower coverage in multiple pools, where the median coverage was 68X across these 16 exons. Due to these concerns with sequence quality, we chose to Sanger sequence all 100 IS individuals for all 13 variants to confirm the variant frequency in our population.

### Statistical comparisons

The Fisher’s exact test (two-sided) was used to calculate the *P*-values for comparisons between the IS population(s) and the 1000 Genomes European population or the EA-ESP, with α = 0.05. Mutalyzer 2.0.beta-31 was used to generate and check all nucleotide and protein positions (Wildeman *et al.* 2008), and positions were verified using MutationTaster and the public version of the Human Gene Mutation Database (HGMD) (Schwarz *et al.* 2014; Stenson *et al.* 2014).

## Results

### Exome sequencing identifies an *HSPG2* variant in an IS family

**Figure 1:**
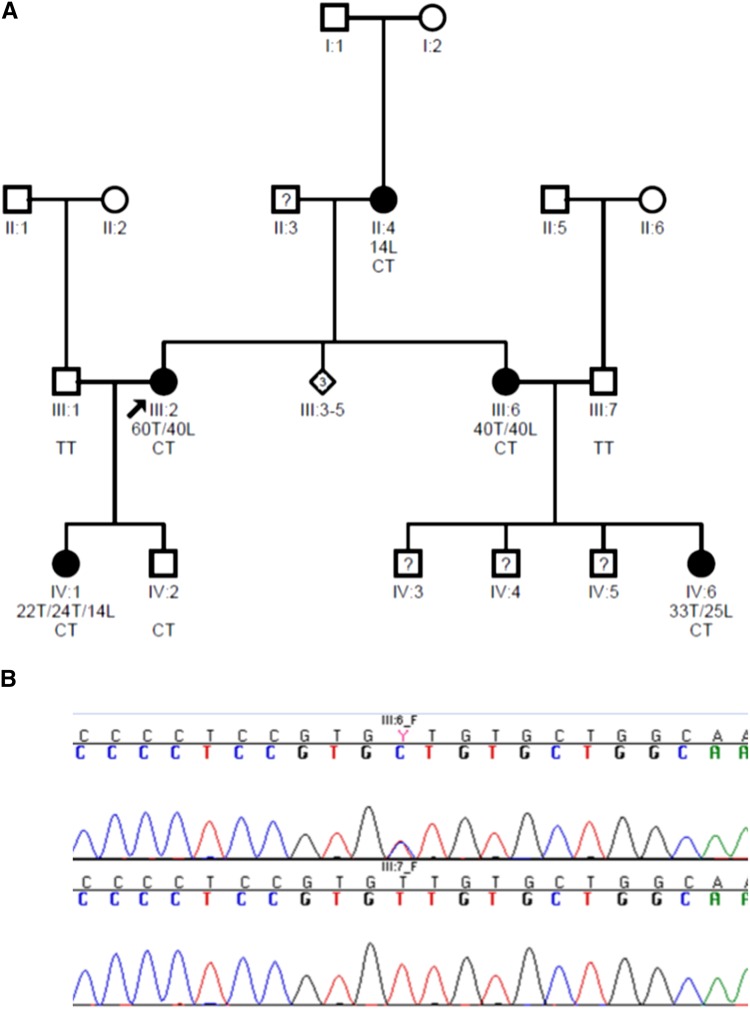
(A) Pedigree for the multigenerational family with IS. Individuals IV:1, IV:6, and II:4 underwent exome sequencing. Sanger sequencing was completed for those three individuals in addition to the affected individuals III:2 and III:6 and unaffected individuals III:1, III:7, and IV:2. Lumbar and thoracic curves are abbreviated with L and T, respectively. Double curves are represented by a slash (right thoracic/left lumbar), and triple curves are represented with three slashes (left high thoracic/right thoracic/left lumbar). CT or TT is the individual genotype at *HSPG2* position p.Asn786Ser. A “?” represents individuals that have not been examined, so their scoliosis status is unknown. (B) Chromatograms for Variant p.Asn786Ser. Chromatograms for p.Asn786Ser are shown for two individuals from the multigenerational family (III:6, affected, and III:7, unaffected). The variant p.Asn786Ser, in the *HSPG2* gene, is heterozygous in the affected individuals within the family, but not in the unaffected individuals (with the exception of IV:2, as discussed in the section *Results*).

We studied a multigenerational family affected with familial IS and of European ancestry (Figure 1A). Affected individuals were diagnosed with familial IS based on radiographic findings of a spinal curvature of ≥10°. The proband, individual III:2, was diagnosed at 13 years of age with a right thoracic curve and a left lumbar curve of 60° and 40°, respectively, and underwent posterior spinal instrumentation and fusion. The transmission pattern in this family suggested a Mendelian mode of inheritance, for which exome sequencing is ideally suited. Exome sequencing, followed by variant detection and filtering, was completed for three individuals in this family. Sequencing coverage was 64× overall, with IV:1, IV:6, II:4 at 97X, 82X, and 22X respectively. Filtering resulted in a list of 16 variants that were at a frequency of <1% in the 1000 Genomes data, were predicted to be damaging (according to the criteria defined in the *Materials and Methods* section and File S1), and were insertions/deletions, nonsense, missense, or splice-site changes. In addition, to be considered for further study, variants were required to be present in all three affected individuals in the family.

After the filtering process, 16 potentially damaging and rare variants were identified in this family (Table 1 and Table S2). One variant was removed due to an excessive frequency in the EA-ESP dataset (in *HGC6.3*, 23% frequency in EA-ESP). For each of the remaining 15 variants, Sanger sequencing was completed for the individuals who originally underwent exome sequencing and for additional relatives with DNA samples [two affected and three unaffected (two of the unaffected individuals were marry-in relatives)]. Sanger sequencing was unable to replicate the original result for 1 of the 15 variants (*OPRD1*) in the original three exome sequenced individuals (Table S3).

**Table 1:** Rare variants (MAF 0.01)*^a^* identified in our multigenerational family with IS*^b^*

Chr	Position	Gene	rs #	Ref	Var	HGVS Protein Position	Variant Type	1000 Genomes Frequency	EA-ESP Frequency
Chr1	22205601	*HSPG2*	rs143736974	T	C	p.N786S	Missense	0.0018	0.009
Chr1	23233346	*EPHB2*	rs28936395	G	A	p.D679N	Missense	0.0023	0.0071
Chr1	28060600	*FAM76A*	rs147811051	G	A	p.R121H	Missense		0.00081
Chr1	29189481	*OPRD1**^c^*	rs370180812	G	T	p.A269S	Missense		0.00017
Chr1	45524416	*ZSWIM5*	rs149382804	G	A	p.R373W	Missense	0.00046	0.0014
Chr1	51873967	*EPS15*	rs61753384	G	A	p.S438L	Missense	0.0064	0.022
Chr4	77662160	*SHROOM3*	rs368255851	A	T	p.D945V	Missense		0.00012
Chr6	43483718	*YIPF3*		GCAT	G	p.D65del	Nonframeshift deletion		0.0095
Chr6	168376925	*HGC6.3**^d^*	rs35001101	G	GT	p.T137Hfs*7	Frameshift insertion		0.24
Chr7	139281560	*HIPK2*		T	TCTGCCG	p.Q873_T874insRQ	Nonframeshift insertion		
Chr7	149473614	*SSPO*	rs117048984	A	G	p.Y77C	Missense		0.04
Chr7	150934827	*CHPF2*	rs144589067	G	A	p.R460Q	Missense	0.0018	0.0039
Chr8	132051970	*ADCY8*	rs75246765	C	A	p.A204S	Missense	0.006	0.016
Chr8	133883671	*TG*	rs114322847	C	T	p.P118L	Missense	0.0087	0.022
Chr10	112570222	*RBM20*		T	TGAG	N/A	Splicing		0.0027
ChrX	132092485	*HS6ST2*	rs181526961	G	A	p.S49L	Missense	0.006	0.0097

MAF, minor allele frequency; IS, idiopathic scoliosis; Chr, chromosome; Ref, reference allele; Var, variant allele; EA-ESP, European American Exome Sequencing Project.

a The original MAF filter used the 1000 genomes frequency.

b Additional details for each variant are provided in Table S2.

c Variant in the *OPRD1* gene was not present in all of the affected individuals in the family and was removed from further analyses.

d Variant in the *HGC6.3* gene was removed from further analyses due to the high frequency in the EA-ESP population.

After filtering and Sanger sequencing, there were 14 variants of interest (Table 1). Therefore, we surveyed the literature for mouse and human phenotypic associations. Previously reported mouse phenotypes for loss-of-function mutations in 13 of the 14 genes produced either phenotypes not clearly associated with musculoskeletal disease or had no evident phenotype. Those 13 genes also have no reported association with a human musculoskeletal phenotype.

The single remaining variant is NM_005529.5:p.Asn786Ser, a heterozygous missense variant in the *HSPG2* gene (MIM *142461) on chromosome 1p36. All of the affected individuals in this family are heterozygous for this variant (CT genotype) (Figure 1B). Of the three unaffected individuals, two are homozygous for the reference allele (TT genotype) and one is heterozygous (IV:2, CT genotype). The presence of the CT genotype in an unaffected male may be due to incomplete penetrance of the IS phenotype.

*HSPG2* encodes for heparan sulfate proteoglycan 2, or perlecan. Perlecan is a key component of basement membranes and is strongly associated with musculoskeletal development, both in mouse and in human (Costell *et al.* 1999; Nicole *et al.* 2000; Arikawa-Hirasawa *et al.* 2001b; Stum *et al.* 2006; Rodgers *et al.* 2007), making it an excellent candidate gene for IS.

### Sequencing of the *HSPG2* gene in 100 individuals with IS

Seeking to further our knowledge of the importance of the *HSPG2* gene in IS, we designed an *HSPG2* sequencing project. Exons from the *HSPG2* gene (97 exons, 14,369 base pair mRNA, 4391 amino acids) were sequenced in 100 unrelated individuals with IS (Denver dataset, curvature range 18−119°) with a custom targeted gene approach on an Illumina MiSeq platform. After variant filtering using the bioinformatic pipeline described previously, we found 14 potentially damaging and rare *HSPG2* variants. Again, variants were defined as potentially damaging according to our criteria defined in the Materials and Methods section and File S1, and rare if the frequency was <1% in the 1000 Genomes database. We were unable to confirm one variant with Sanger sequencing, and it was consequently removed from further analyses (see the section *Materials and Methods*). Each variant had a frequency of <1% in the 1000 Genomes database, and 3 of the 13 identified variants were unique (not previously identified in the 1000 genomes data or in the NHLBI ESP; Table 2 and Table S4). All 13 variants were tested by Sanger sequencing in all 100 individuals to assess variant frequencies in our IS population. Including one individual from our multigenerational family (IV:1), we had a total of 101 individuals with 20 occurrences of *HSPG2* variants. One individual had two *HSPG2* variants.

**Table 2:** *HSPG2* rare variants (MAF <0.01)*^a^* Identified in individuals with IS*^b^*

Position	rs #	Ref	Var	HGVS Position*^c^*	Variant Type	1000 Genomes Frequency	EA-ESP Frequency	Denver (N = 101)	St. Louis (N = 140)	IS Allele Frequency
22149967	rs145687082	C	T	p.V4340M	Missense		0.0014	1		0.0021
22150639	rs141280063	C	T	p.E4292K	Missense		0.00047	1		0.0021
22150854		C	T	p.G4254S	Missense			1		0.0021
22154901	rs146179360	G	A	p.R4086W	Missense		0.0012		1	0.0021
22155345	rs140139732	G	A	p.R4074C	Missense		0.0014	1	1	0.0041
22155455	rs143015575	C	A	p.R4037L	Missense		0.00082		1	0.0021
22155960		C	T	p.G3970R	Missense			1		0.0021
22159039	rs78280998	C	T	p.R3719Q	Missense				1	0.0021
22161394	rs143543800	C	T	p.V3500M	Missense	0.0018	0.0041	1		0.0021
22168855	rs114851469	G	A	p.R2977W	Missense	0.0032	0.012		5	0.010
22169325	rs41266007	C	T	p.G2950R	Missense	0.0078	0.02	3	4	0.015
22182115	rs2229474	G	A	p.R1919C	Missense	0.0041	0.0087	1	2	0.0062
22182333	rs140954748	G	A	p.A1883V	Missense		0.0012		1	0.0021
22186669		C	T	N/A	Splicing			1		0.0021
22188289	rs142433309	G	A	p.T1639M	Missense	0.0009	0.0036	2 (1 has AA)	1	0.0083
22188328	rs41311989	C	T	p.R1626H	Missense	0.006	0.0011	1	1	0.0041
22202483	rs62642528	G	A	p.P1019L	Missense		0.014	2	1	0.0062
22205601	rs143736974	T	C	p.N786S	Missense	0.0018	0.009	4	6	0.021
22206959		A	G	p.Y698H	Missense				1	0.0021
22206977	rs143669458	C	T	p.V692M	Missense	0.00092	0.0033		1	0.0021
22211941	rs75467696	G	T	p.T361N	Missense	0.0023	0.0066		1	0.0021

MAF, minor allele frequency; IS, idiopathic scoliosis; Ref, reference allele; Var, variant allele EA-ESP, European American Exome Sequencing Project.

a The original MAF filter used the 1000 genomes frequency.

b Additional details for each variant are provided in Table S4.

c *HSPG2* HGVS positions use NM_005529.

One of the 13 variants was the same variant previously identified from the exome sequencing of our multigenerational IS family (p.Asn786Ser). This specific variant was heterozygous in 3 of the 100 additional sequenced individuals. In the 1000 Genomes data, this variant was only found in four individuals, all male (three from the European subset and one from the Puerto Rican subset). This difference is significant, with the CT genotype present in 4 of 101 IS individuals compared with 3 of 379 1000 Genomes European individuals [*P* = 0.039, odds ratio (OR) 5.2, 95% confidence interval (95% CI) 0.96−30]. However, the difference is not significant when we compare with the EA-ESP dataset (77/4300 individuals, *P* = 0.114, OR 2.3, 95% CI 0.69−6.6). This is potentially related to the small number of individuals sequenced in our study (N = 101 individuals) compared with the EA-ESP database (N = 4300).

In summary, in the Denver IS dataset, we identified 13 potentially damaging and rare variants in the *HSPG2* gene, with 20 occurrences in 101 individuals with IS. This finding provides further support that *HSPG2* may have an important role in IS etiology.

### Further investigation of the *HSPG2* finding in an independent IS population

Next, we investigated *HSPG2* damaging variants in the exome sequencing data from an independent group of 140 IS individuals (St. Louis dataset). We identified 7 of the original 13 Denver *HSPG2* variants in this St. Louis dataset, including p.Asn786Ser. The CT genotype for this variant is present in six individuals in the St. Louis dataset (compared with 77/4300 EA-ESP, *P* = 0.046, OR = 2.5, 95% CI 0.95-6.0). Furthermore, an additional 11 potentially damaging variants in *HSPG2* were discovered that were not previously identified in the Denver IS group, for a total of 18 *HSPG2* variants in the St. Louis dataset (Table 2 and Table S4). We tested all 18 variants by Sanger sequencing and found that three low-quality variants did not confirm, resulting in 15 verified *HSPG2* variants from the St. Louis dataset. One individual had two *HSPG2* variants.

Collectively, in the combined Denver-St. Louis IS population, the data for the p.Asn786Ser variant are compelling. There are 10 IS individuals with a CT genotype of 241 individuals total. We compared this with 77 of 4300 in the EA-ESP population and found that the CT genotype is significantly enriched in the combined IS population (*P* = 0.024, OR 2.4, 95% CI 1.1−4.8).

**Figure 2:**
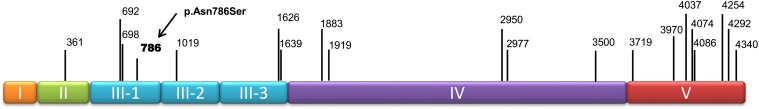
Rare *HSPG2* variants identified in IS patients. Structure of the HSPG2 protein showing the amino acid positions of the rare coding variants identified in the combined IS cohort of 241 individuals. Amino acid positions are based on transcript NM_005529.5. Variant p.Asn786Ser is the original variant identified in this study (combined Denver−St. Louis dataset, *P* = 0.024, odds ratio 2.4). Also note that variant 22186669 (c.5014+1G > A) is not shown on the diagram because it is a splicing variant and does not have an amino acid position. IS, idiopathic scoliosis.

Overall, 21 potentially damaging and rare variants in *HSPG2* were identified in the combined Denver-St. Louis IS population, with 48 occurrences in 241 individuals. The variants are distributed across the *HSPG2* gene (Figure 2).

## Discussion

We performed exome sequencing for a multigenerational family with familial IS and identified a potentially damaging and rare variant in the *HSPG2* gene. Our previous linkage results were reviewed for potential correlation with the location of newly identified variants (Miller *et al.* 2005, 2006; Alden *et al.* 2006; Clough *et al.* 2010; Marosy *et al.* 2010). When this particular family was analyzed individually, marginal linkage signals were noted at nine loci (Miller, unpublished data, 2014). One of these loci, located on chromosome 1 (19−48.3 Mb), contains the *HSPG2* gene. These ancillary linkage data provide additional evidence that a variant within *HSPG2* potentially contributes to the IS phenotype in this family.

Upon further sequencing of the *HSPG2* gene in an additional 100 individuals with IS, we identified 13 potentially damaging and rare variants in *HSPG2*, with 20 occurrences in 101 individuals. Finally, using exome sequencing data from a second IS population, we found 15 variants in *HSPG2*. Combined, we found 21 rare variants with 48 occurrences in 241 individuals with IS. Specifically, p.Asn786Ser was present in 10 individuals in our dataset and is enriched in these two IS populations when compared with the EA-ESP dataset (*P* = 0.024, OR 2.4).

*HSPG2* encodes for heparan sulfate proteoglycan 2, or perlecan. Perlecan is a modular proteoglycan consisting of five structural core domains to which long chains of glycosaminoglycans (GAGs: heparan sulfate or chondroitin sulfate) are attached (Figure 2) (Murdoch *et al.* 1992). Domain I, the N-terminal domain, is unique to perlecan and has three attachment sites for heparan sulfate/chondroitin sulfate chains. Domain II is homologous to low-density lipoprotein (LDL) receptor sites. Domain III consists of multiple modules homologous to laminin alpha chains and laminin epidermal growth factor-like (EGF-like) repeats and is divided into three subdomains (III-1, III-2, III-3) (Schulze *et al.* 1996). Fibroblast growth factor-7 (FGF-7), platelet-derived growth factor (PDGF), and von Willebrand A-domain related protein (WARP) are known to bind subdomains III-1, III-2, and III-2, respectively (Gohring *et al.* 1998; Mongiat *et al.* 2000; Allen *et al.* 2006). The largest domain, domain IV, contains multiple immunoglobulin G−like (IgG-like) repeats similar to neural cell adhesion molecules. Domain V, at the C-terminus of the protein, has homology to the globular domain of laminin and contains a GAG attachment and EGF-like repeats. This domain interacts with cell-surface integrins (α2β1), forming additional complexes linking the extracellular matrix (ECM) with the cell, and is responsible for perlecan self-assembly (Bix *et al.* 2004).

Perlecan stabilizes the ECM and is localized to basement membranes, vascular structures, cartilage, and osteogenic tissues (Costell *et al.* 1999). This protein is abundantly expressed by skeletal myofibers and is incorporated into the basal lamina of both the synaptic and extrasynaptic regions within the neuromuscular junction (NMJ) (Singhal and Martin 2011). Perlecan binds multiple proteins and signaling molecules {including FBN1 [previously implicated in IS (Buchan *et al.* 2014)], FGF-7, latent transforming growth factor-beta binding protein-2 (LTBP-2)}, indicating it has a role as an ECM signaling scaffold with an ability to participate in cellular proliferation, differentiation, and migration (Arikawa-Hirasawa *et al.* 1999; Farach-Carson and Carson 2007; Hayes *et al.* 2011; Hayes *et al.* 2014). Perlecan is strongly associated with musculoskeletal development because it is essential for endothelial growth, regeneration, and both avascular cartilage and skeletal development, making *HSPG2* an excellent gene candidate for IS.

Classically, pathogenic mutations in the *FBN1* gene result in Marfan syndrome, a multisystem disorder that includes the musculoskeletal abnormality of scoliosis. Early investigations noted FBN1 abnormalities in the paraspinal tissues and fibroblasts of select individuals with severe IS (Miller *et al.* 1996). Most recently, Buchan *et al.* (2014) identified rare variants in the *FBN1* gene as risk factors for severe spinal deformity in individuals with IS (Buchan *et al.* 2014). Collectively, these findings and the known colocalization of perlecan and FBN1 within paraspinal tissues make *HSPG2* an excellent candidate gene for IS pathogenesis.

Mutations within the *HSPG2* gene have been associated with two human diseases transmitted in an autosomal recessive pattern of inheritance, Schwartz-Jampel syndrome, type 1 (SJS1) [MIM: #255800] and dyssegmental dysplasia Silverman-Handmaker type (DDSH) [MIM: #224410]. Individuals with SJS1 are characterized by a skeletal dysplasia, including kyphoscoliosis, joint contractures, and myotonia resulting in a fixed facial expression and prolonged muscle contractions (Nicole *et al.* 2000; Stum *et al.* 2006). SJS1 mutations occur throughout the *HSPG2* gene and are not concentrated in a specific exon or domain (Table S5) (Nicole *et al.* 2000; Arikawa-Hirasawa *et al.* 2002; Stum *et al.* 2006; Rodgers *et al.* 2007). In SJS1, the mutated proteins have varying degrees of functionality. Animal models have shown site-specific mutations of the gene, which result in down-regulation of perlecan at the transcriptional level (Rodgers *et al.* 2007). In contrast, DDSH is a more severe phenotype than SJS1 and is considered lethal (Arikawa-Hirasawa *et al.* 2001b). The causative mutational events for DDSH are functional null mutations, resulting in a truncated perlecan protein core that is not secreted (Table S5) (Arikawa-Hirasawa *et al.* 2001a). This is similar to the *Hspg2* homozygous knockout mouse, which exhibits significant kyphoscoliosis and skeletal defects (Costell *et al.* 1999). Thus, homozygous *HSPG2* mutations in humans and mice display a more severe phenotype with an earlier onset compared with the adolescent-onset FIS phenotype in individuals with heterozygous *HSPG2* variants. This finding suggests that haploinsufficiency may result in a later onset and progressive disease, as observed in FIS.

Similar to SJS1 mutational events, the variants we identified in IS individuals are spread out across the *HSPG2* gene. Our original variant (p.Asn786Ser) is located in domain III-1, which is known to bind FGF-7 (Figure 2). One IS individual has a damaging variant (22202483, p.Pro1019Leu) at a base position also identified in SJS1 individuals (Stum *et al.* 2006). However, this variant has an allele frequency of 1.3% in the EA-ESP and thus is unlikely to be solely causative for SJS1, based on a predicted homozygote frequency of 0.018%.

We completed a test of variant burden across the entire *HSPG2* gene, comparing the occurrence of potentially damaging and rare variants in individuals with IS to the EA-ESP control population. We used a Z test for population proportions to compare the number of variant occurrences in the two populations. Using the overall combined dataset, we found there were 48 occurrences in 241 individuals with IS compared with 720 occurrences in 4300 individuals in the EA-ESP dataset (*P* = 0.20). If we remove the variants in perlecan domain IV from both datasets, because domain IV is highly repetitive and the functions are more likely to be redundant within the gene, there are 31 occurrences in 241 individuals with IS compared with 385 occurrences in 4300 individuals in the EA-ESP dataset (*P* = 0.040). This marginally significant result increases our confidence that variants in *HSPG2* contribute to the IS phenotype, but sequencing in additional individuals with IS will be needed to draw strong conclusions.

IS, defined as a lateral spinal curvature of at least 10°, is present in 3% of the general population and can lie undetected within many individuals. Thus, a variant that contributes to the IS phenotype could be expected to be present in a control population at a reasonably high frequency. In addition, IS is characterized by incomplete penetrance, so not all individuals who carry a variant are expected to have clinical disease, as seen with individual IV:2, who is unaffected but is heterozygous at the variant of interest (p.Asn786Ser). Finally, we do not expect that one gene is causative for IS in all individuals. We believe that *HSPG2* may contribute to the IS phenotype in a subset of individuals with IS but that other genes may be required to modify the disease and result in clinical/identifiable disease (*i.e.*, another variant in a different gene may also be required to have a larger/progressive curvature). Future studies should include both sequencing the *HSPG2* gene in larger IS cohorts and performing functional studies of the identified variants to provide confirmation that the variants contribute to the IS phenotype.

In summary, IS is a complex disease. Genetic findings have been hampered by incomplete penetrance, subclinical disease in control populations, unknown modes of inheritance, and genetic heterogeneity. Additionally, IS may be a polygenic disease, even within families. By demonstrating the presence of the p.Asn786Ser variant in all affected individuals in a multigenerational family, with incomplete penetrance in one unaffected individual, and enrichment of this variant in two independent cohorts of IS patients, we now have evidence that rare variants in the *HSPG2* gene potentially contribute to IS susceptibility.

## Supplementary Material

Supporting Information
