# Supplementary material for: Exome Sequencing Identifies a Rare HSPG2 Variant Associated with Familial Idiopathic Scoliosis
Source: G3 (Bethesda). 2014 Dec 12;5(2):167–74. doi: 10.1534/g3.114.015669 (PMC4321025; doi:10.1534/g3.114.015669)
Supplement: Supporting Information [file supp_5_2_167__index.html]

Exome Sequencing Identifies a Rare HSPG2 Variant Associated with Familial Idiopathic Scoliosis — Supporting Information 

# Exome Sequencing Identifies a Rare *HSPG2* Variant Associated with Familial Idiopathic Scoliosis

## Supporting Information for Baschal *et al.*, 2015

**Files in this Data Supplement:**

- File S1 - Supplemental Methods (PDF, 154 KB)
- Table S1 - Primers used in this study. (.xlsx, 15 KB)
- Table S2 - Additional details for the 16 variants identified in our multigenerational family with IS. (.xlsx, 15 KB)
- Table S3 - Sanger sequencing results for 15 variants in our multigenerational family with IS. (.xlsx, 11 KB)
- Table S4 - Additional details for the 21 *HSPG2* variants identified in individuals with IS. (.xlsx, 13 KB)
- Table S5 - *HSPG2* mutations reported in individuals with either SJS1 or DDSH. This is not a comprehensive list, but represents the majority of reported *HSPG2* mutations. (.xlsx, 13 KB)
