# Supplementary material for: Exome Sequencing Identifies a Rare HSPG2 Variant Associated with Familial Idiopathic Scoliosis
Source: G3 (Bethesda). 2014 Dec 12;5(2):167–74. doi: 10.1534/g3.114.015669 (PMC4321025; doi:10.1534/g3.114.015669)
Supplement: Supporting Information [file supp_g3.114.015669_015669SI.pdf]

G3 · Genes | Genomes | Genetics

Exome sequencing identifies a rare *HSPG2* variant associated with familial idiopathic scoliosis

Erin E. Baschal[*], Cambria I. Wethey[*], Kandice Swindle[*], Robin M. Baschal[§], Katherine Gowan[†], Nelson L.S. Tang[‡], David M. Alvarado**, Gabe E. Haller**, Matthew B. Dobbs[**,§§], Matthew R.G. Taylor[††], Christina A. Gurnett[**,‡‡], Kenneth L. Jones[†], Nancy H. Miller[*,§]

* Department of Orthopedics, University of Colorado Denver Anschutz Medical Campus, Aurora, Colorado, 80045

§ Musculoskeletal Research Center, Children's Hospital Colorado, Aurora, Colorado, 80045

† Department of Biochemistry, University of Colorado Denver Anschutz Medical Campus, Aurora, Colorado, 80045

‡ Department of Chemical Pathology and Li Ka Shing Institute of Health Sciences, Faculty of Medicine, The Chinese University of Hong Kong, Hong Kong SAR.

** Department of Orthopaedic Surgery, Washington University School of Medicine, St. Louis, Missouri, 63130

§§ Saint Louis Shriners Hospital for Children, St. Louis, Missouri, 63131

†† Department of Cardiology, University of Colorado Denver Anschutz Medical Campus, Aurora, Colorado, 80045

‡‡ Department of Neurology, Washington University School of Medicine, St. Louis, Missouri, 63130

IRB APPROVAL: Colorado Multiple Institutional Review Board (COMIRB), Study Approval # 06-1161 and 07-0417

Correspondence:

*Nancy H. Miller, University of Colorado Denver Anschutz Medical Campus, 12800 E. 19th Ave., MS8343, Aurora, CO 80045

Tel: 1-303-724-0046, Fax: 1-303-734-0394, email: Nancy.Hadley-Miller@ucdenver.edu

DOI: 10.1534/g3.114.015669

File S1

SUPPLEMENTAL METHODS

Bioinformatics Filtering

dbNSFP identifies prediction scores from SIFT, Polyphen2, LRT, Mutation Taster, and Mutation Assessor, and conservation scores from GERP++ and PhyloP [1]. Nonsense and missense variants were considered damaging if two or more of the following criteria were met:

- SIFT score < 0.5
- Polyphen2_HDIV_pred prediction of D
- LRT_pred prediction of D
- MutationTaster_pred prediction of A or D
- MutationAssessor_pred prediction of high or medium

A variant could still be considered damaging with only one criterion met, if 3 or less of the algorithms reported a score. Splice variants and insertions/deletions were automatically considered damaging.

PCR

Primers were designed using the program Primer3 [2-3]. Primer sequences can be found in **Supplemental Table 1**. PCR was performed in a 20 µl reaction mix containing 40 ng of genomic DNA, a final concentration of 1X MasterAmp™ PCR Premix D (Epicentre, Madison, WI), 1 µM of forward and reverse primers (Integrated DNA Technologies, Coralville, IA) and 1 unit of Taq polymerase (Sigma-Aldrich, St. Louis, MO, catalog number D6677). Touchdown PCR was completed as described: one cycle of 94°C for 3 min, 35 cycles of touchdown PCR (94°C for 15 sec, touchdown annealing temperature for 1 min, and 72°C for 1 min), 25 cycles of 94°C for 15 sec, 55°C for 1 min, and 72°C for 1.5 min, followed by one cycle at 72°C for 15 min. For touchdown cycles, the annealing temperature was 65°C initially, and was decreased by 0.3 degrees each cycle, ending at 55°C. Agarose gel electrophoresis was used to confirm PCR products, and all primer sets produced a single band.

Sanger Sequencing

Sanger sequencing was completed at either the University of Colorado Denver Barbara Davis Center for Childhood Diabetes or at Beckman Coulter Genomics. At the Barbara Davis Center, PCR fragments were sequenced at the Molecular Biology Core Facility following ExoSAP-IT® treatment (Affymetrix, Cleveland, OH) per manufacturer protocol. Treated fragments (template)

were diluted 1:10 in preparation for Sanger sequencing, which was performed using BigDye3.1 chemistry on a 3130xl instrument (Life Technologies, Grand Island, NY, USA).

At Beckman Coulter Genomics, PCR products were purified using Agencourt AMPureXL SPRI technology PCR cleanup system (Agencourt, Bevery, MA, USA). Sequencing reactions utilized Applied Biosystems BigDye® Version 3.1 and were purified using Agencourt CleanSeq® dye-terminator removal kit. Products were then Sanger sequenced on an Applied Biosystems 3730xl DNA Analyzer (Applied Biosystems, Foster City, CA, USA).

REFERENCES

1. Liu X, Jian X, Boerwinkle E: Dbnsfp: A lightweight database of human nonsynonymous snps and their functional predictions. Hum Mutat 2011;32:894-899.
2. Koressaar T, Remm M: Enhancements and modifications of primer design program primer3. Bioinformatics 2007;23:1289-1291.
3. Untergasser A, Cutcutache I, Koressaar T, Ye J, Faircloth BC, Remm M, Rozen SG: Primer3--new capabilities and interfaces. Nucleic Acids Res 2012;40:e115.

Tables S1-S5

Available for download as Excel files at http://www.g3journal.org/lookup/suppl/doi:10.1534/g3.114.015669/-/DC1

Table S1: Primers used in this study

Table S2: Additional details for the 16 variants identified in our multigenerational family with IS

Table S3: Sanger sequencing results for 15 variants in our multigenerational family with IS

Table S4: Additional details for the 21 *HSPG2* variants identified in individuals with IS

Table S5: *HSPG2* mutations reported in individuals with either SJS1 or DDSH. This is not a comprehensive list, but represents the majority of reported *HSPG2* mutations.
